# A Study on the Accuracy of Point-of-Care Ultrasound in the Diagnosis and Management of Necrotizing Fasciitis

**DOI:** 10.7759/cureus.83814

**Published:** 2025-05-09

**Authors:** Eswar Medikonda, Aravind Patil, Rajashekhar Muchchandi, Manjunath Kotennavar, Pradeep P Jaju, Sanjeev Rathod, Manjunath S Savant, Veena Ghanteppagol, Saket P Shetty, Divyang GB, Shreeya Doddannavar, Smit Parikh

**Affiliations:** 1 General Surgery, Shri B M Patil Medical College Hospital and Research Centre, Bijapur Lingayat District Educational (BLDE) (Deemed to be University), Vijayapura, IND; 2 Surgery, Shri B M Patil Medical College Hospital and Research Centre, Bijapur Lingayat District Educational (BLDE) (Deemed to be University), Vijayapura, IND; 3 Radiodiagnosis, Shri B M Patil Medical College Hospital and Research Centre, Bijapur Lingayat District Educational (BLDE) (Deemed to be University), Vijayapura, IND

**Keywords:** clinical outcomes, diagnostic accuracy, laboratory risk indicator for necrotizing fasciitis (lrinec) score, mortality, necrotizing fasciitis, point-of-care ultrasound, soft tissue infection, surgical debridement

## Abstract

Background

Necrotizing fasciitis (NF) is a rapidly progressive, life-threatening soft tissue infection with high mortality rates. Early diagnosis and prompt surgical intervention are crucial for survival, yet the initial diagnosis remains challenging due to nonspecific early presentations. This study evaluated the diagnostic accuracy of point-of-care ultrasound (POCUS) in identifying NF and its utility in guiding clinical management decisions.

Methods

This prospective observational study included 85 patients with suspected NF at a tertiary care center in India from April 2023 to April 2025. Trained emergency physicians performed POCUS examinations using high-frequency linear transducers and low-frequency curvilinear transducers when necessary. Sonographic findings were documented and correlated with surgical observations, clinical outcomes, and laboratory parameters. Primary outcomes included POCUS diagnostic accuracy, the need for surgical intervention, and mortality rates.

Results

The study population had a mean age of 50.2 years, with male predominance (62.4%) and primarily lower limb involvement (77.6%). POCUS demonstrated high positivity (97.6%) with predominantly fluid collection (77.6%), the loss of vascularity (65.9%), and fascial thickening (52.9%). Sensitivity was highest for fascial thickening (97.1%) and fluid collection (92.5%). Most required multiple debridements (83.5%), with 43.5% undergoing three procedures. At three-week follow-up, 25.9% achieved partial recovery and 18.8% complete recovery, with 15.3% mortality. Complications included amputation (11.8%), sepsis (9.4%), and wound infection (8.2%). POCUS assessment at three weeks showed persistent changes in 36.5% of patients despite clinical improvement in many cases.

Conclusion

POCUS is a highly sensitive diagnostic tool for NF with excellent correlation to surgical findings. Its immediate availability, non-invasive nature, and repeatability position it as a valuable adjunct in the initial assessment and monitoring of this life-threatening condition. The integration of POCUS into standard assessment protocols for suspected NF can expedite diagnosis, guide surgical interventions, and improve clinical outcomes.

## Introduction

Necrotizing fasciitis (NF) is a rare but potentially lethal soft tissue infection, with an incidence ranging from 0.3 to five cases per 100,000 people. It is characterized by the rapidly progressive necrosis of the fascia and subcutaneous tissue, with reported mortality rates between 25% and 75% despite advances in modern medical care [1]. Early recognition and immediate surgical intervention are critical determinants of survival; however, initial diagnosis remains challenging due to the often subtle and nonspecific nature of early clinical presentations [2].

Traditionally, diagnosis has relied on clinical assessment, laboratory markers, and computed tomography (CT) or magnetic resonance imaging (MRI). However, these imaging modalities may not be readily available in emergency settings, and the time required to obtain them can delay crucial therapeutic interventions [3]. Furthermore, patient transportation for advanced imaging studies may be problematic, particularly in hemodynamically unstable cases [4].

Point-of-care ultrasound (POCUS) has emerged as a promising diagnostic tool in emergency medicine, offering real-time, bedside evaluation of soft tissue infections [5]. Technological advances in portable ultrasound devices have significantly improved image quality and diagnostic capabilities [6]. The potential advantages of POCUS in NF diagnosis include its non-invasive nature, the lack of ionizing radiation, cost-effectiveness, and, most importantly, its ability to provide immediate results at the patient’s bedside [7].

Several sonographic features have been described in NF, including fascial thickening, subcutaneous fluid accumulation, and gas in soft tissues [8]. Fascial thickness greater than 8 mm has been suggested as a significant diagnostic marker for NF [6]. However, the diagnostic accuracy of POCUS in NF and its impact on clinical decision-making and patient outcomes require further systematic evaluation [9]. Furthermore, the role of POCUS in tracking disease progression and shaping surgical management strategies remains an area ripe for deeper exploration and understanding [10].

This study aims to evaluate the diagnostic accuracy of POCUS in identifying necrotizing fasciitis, using intraoperative surgical findings as the reference standard. We further seek to compare POCUS results to final clinical outcomes and findings from other imaging modalities, and to assess its impact on time to diagnosis, surgical planning, and patient outcomes.

## Materials and methods

This prospective observational open-label study was conducted at the Department of General Surgery, Shri B M Patil Medical College Hospital and Research Centre, Vijayapura, from April 2023 to April 2025.

The Ethical Committee of Bijapur Lingayat District Educational (BLDE) (Deemed to be University) approved the study on 10-04-2024 (ethical approval number: BLDE(DU)/IEC/924/2023-24).

Eighty-five patients aged 18-80 years with clinically suspected necrotizing fasciitis (NF) were included in the study. Patients with peripheral vascular disease, a history of prior NF treatment, nonhealing ulcers persisting for more than six months, or traumatic injuries were excluded. The sample size was calculated to be 85 based on a 95% confidence level and 5% absolute precision.

All eligible patients underwent a comprehensive clinical examination upon admission. Vital parameters were monitored, and blood samples were collected for complete blood count, renal and liver function tests, coagulation profile, blood glucose levels, and inflammatory markers, including C-reactive protein (CRP) and procalcitonin. Blood cultures were obtained before initiating empirical antibiotic therapy.

Trained emergency physicians, blinded to patients’ clinical and laboratory findings and final diagnoses, performed and interpreted point-of-care ultrasound (POCUS) examinations using high-frequency linear transducers (7-15 MHz) and low-frequency curvilinear transducers (2-5 MHz) when deeper tissue evaluation was required. Fascial thickening was identified and recorded as a key sonographic feature, with thickening defined as greater than 8 mm. Fascial thickness measurements were taken using the real-time imaging provided by the ultrasound, and comparisons were made to intraoperative findings documented by the surgical team. Intraoperative fascial thickening was assessed by visual inspection and, if necessary, sterile measurement using a caliper. The affected regions were systematically scanned, and specific sonographic findings were documented, including fascial thickening and echogenicity, subcutaneous tissue involvement, the presence of fluid collections, gas in soft tissues, and the depth of tissue involvement. POCUS findings were used to delineate the extent of tissue involvement for surgical planning, and all images were stored digitally. Surgical exploration, serving as the reference standard, was performed within six hours of the POCUS examination to ensure timely and valid diagnostic comparisons.

Based on POCUS findings and clinical assessment, patients underwent surgical debridement. The surgical team documented the correlation between ultrasound-marked areas and actual tissue involvement during surgery. Post-debridement wound care protocols were standardized for all patients. Patients’ comorbidities were thoroughly evaluated and documented, with management protocols adjusted accordingly.

Primary outcome measures included the accuracy of POCUS in identifying the extent of tissue involvement compared to surgical findings, time from presentation to surgical intervention, the length of hospital stay, need for repeat debridement, and in-hospital mortality. Secondary outcomes included patient condition at discharge, symptom relief, wound healing, progression, functional recovery, and quality of life assessment. Posttreatment follow-up was conducted at regular intervals with the photographic documentation of wound progression.

Data was entered in Excel (Microsoft Corp., Redmond, WA) and analyzed using SPSS version 21 (IBM Corp., Armonk, NY). Quantitative data was presented as mean, median, standard deviation, and ranges, while qualitative data was expressed as frequency and percentages. Student’s t-test was used to test the significance of means, with P values of <0.05 considered significant.

## Results

A total of 85 patients with suspected necrotizing fasciitis (NF) aged 18-80 years were enrolled. Table 1 illustrates the demographic profile and clinical characteristics of the study population. The majority of patients (42.4%) were middle-aged (41-60 years), with a male predominance (62.4%). Lower limbs were the most commonly affected body part (77.6%), followed by upper limbs (20.0%). The mean duration of symptoms before presentation was 7.75 ± 3.89 days, with patients requiring an average hospital stay of 25.08 ± 11.1 days. These findings highlight the typical presentation pattern of NF in our setting and provide context for the subsequent analysis of diagnostic approaches and outcomes.

**Table 1:** Demographic and clinical characteristics of patients (n = 85) SD: standard deviation

Characteristic	n (%) or Mean ± SD
Age distribution	
20-40 years	31 (36.5%)
41-60 years	36 (42.4%)
61-80 years	18 (21.2%)
Gender	
Male	53 (62.4%)
Female	32 (37.6%)
Affected body parts	
Lower limb	66 (77.6%)
Upper limb	17 (20.0%)
Abdomen	2 (2.4%)
Laterality	
Right	46 (54.1%)
Left	39 (45.9%)
Duration of symptoms (days)	7.75 ± 3.89
Length of hospital stay (days)	25.08 ± 11.1

Fever (90.6%) and tachycardia (87.1%) were the most common systemic features, reflecting the profound inflammatory and hemodynamic impact of this severe infection. Local signs suggestive of NF were also carefully documented: limb swelling (81.2%), skin discoloration (62.4%), the presence of bullae or blebs (48.2%), and palpable crepitus (37.6%).

Table 2 presents the key laboratory parameters and laboratory risk indicator for necrotizing fasciitis (LRINEC) scores observed in our cohort. Laboratory investigations revealed marked leukocytosis (mean WBC: 18,505.6 ± 7,391.8 cells/mm³), elevated C-reactive protein (169 ± 68.7 mg/L), and increased creatinine levels (1.82 ± 0.7 mg/dL). Notably, 50.6% of patients had LRINEC scores of >8, indicating high risk for NF. These findings emphasize the inflammatory response and multisystem involvement characteristic of NF and validate the utility of the LRINEC scoring system in our patient population. The mean WBC count was significantly higher in patients with LRINEC scores of >8 compared to those with scores of <5 (20,128.3 ± 6,594.7 versus 15,863.2 ± 5,887.3; t = 3.21; p = 0.002). CRP levels were significantly elevated in patients requiring multiple debridements compared to single debridement (182.4 ± 53.6 versus 128.7 ± 44.9; t = 4.07; p < 0.001).

**Table 2:** Laboratory findings and LRINEC scores SD, standard deviation; CRP, C-reactive protein; LRINEC, laboratory risk indicator for necrotizing fasciitis

Variable	n (%) or Mean ± SD
Laboratory findings	
WBC count (cells/mm³)	18,505.6 ± 7,391.8
CRP (mg/L)	169 ± 68.7
Creatinine (mg/dL)	1.82 ± 0.7
LRINEC score distribution	
<5	35 (41.2%)
6-7	7 (8.2%)
>8	43 (50.6%)

**Figure 1:**
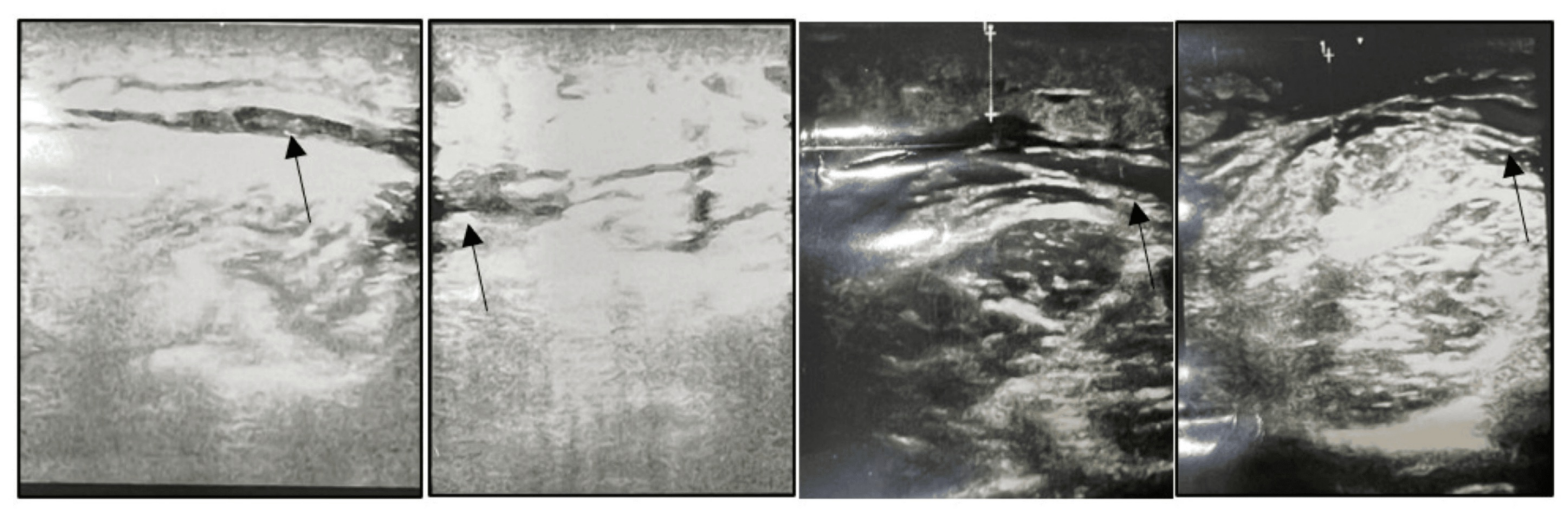
Images showing fluid collection

Table 3 demonstrates the correlation between POCUS findings, surgical observations, and diagnostic accuracy. POCUS demonstrated high positivity (97.6%) with predominant findings of fluid collection (77.6%), the loss of vascularity (65.9%), and fascial thickening (52.9%). The majority of patients required multiple debridements, with 43.5% undergoing three procedures and 40.0% requiring two (Figure 1).

**Table 3:** Diagnostic findings and surgical management POCUS: point-of-care ultrasound

Variable	n (%) or Value
POCUS findings	
POCUS positivity	83 (97.6%)
Fluid collection	66 (77.6%)
Loss of vascularity	56 (65.9%)
Fascial thickening of >8 mm	45 (52.9%)
Surgical findings	
Fluid collection	33 (38.8%)
Subcutaneous gas	20 (23.5%)
Fascial thickening of >8 mm	15 (17.6%)
Number of debridements required	
One	14 (16.5%)
Two	34 (40.0%)
Three	37 (43.5%)

Table 4 shows the diagnostic performance of ultrasound findings in detecting necrotizing fasciitis. The findings demonstrate high sensitivity for fascial thickening (97.1%) and fluid collection (92.5%), with moderate sensitivity for subcutaneous gas (66.2%). Specificity was high across all parameters, with subcutaneous gas exhibiting the highest specificity (88.2%). Positive predictive values (PPV) ranged from 80.7% for fluid collection to 84.9% for subcutaneous gas, while negative predictive values (NPV) were highest for fascial thickening (96.6%) and fluid collection (91.3%). These findings underscore the diagnostic utility of POCUS in NF and highlight the typically aggressive surgical approach needed for effective management.

**Table 4:** Diagnostic accuracy of ultrasound findings PPV, positive predictive value; NPV, negative predictive value

Ultrasound Findings	Sensitivity	Specificity	PPV	NPV
Fascial thickening	97.10%	80%	82.80%	96.6%
Fluid collection	92.50%	77.80%	80.70%	91.30%
Subcutaneous gas	66.20%	88.20%	84.90%	72.30%

Table 5 summarizes the clinical trajectory and outcomes of our patients. At one week post-intervention, 36.5% remained in critical condition, with 30.6% stable and 29.4% improved. By three weeks, 25.9% achieved partial recovery and 18.8% demonstrated complete recovery, while 15.3% had died. Complications included amputation (11.8%), sepsis (9.4%), and wound infection (8.2%), with 63.5% of patients experiencing no complications. POCUS assessment at three weeks showed persistent changes in 36.5% of patients despite clinical improvement in many cases. Patient-reported outcomes were favorable, with 52.9% reporting satisfactory symptom relief scores and 57.6% indicating favorable wound healing scores. Patients with fascial thickening on POCUS had significantly longer hospital stays compared to those without (28.4 ± 9.7 days versus 21.3 ± 8.4 days; t = 3.58; p = 0.001). These findings illustrate the clinical course, mortality, morbidity, and functional outcomes associated with NF in our setting.

**Table 5:** Clinical outcomes and follow-up assessment POCUS: point-of-care ultrasound

Outcome Measure	n (%)
Patient condition at one week	
Critical	31 (36.5%)
Stable	26 (30.6%)
Improved	25 (29.4%)
Deceased	3 (3.5%)
Clinical outcome at three weeks	
Partial recovery	22 (25.9%)
Ongoing treatment	17 (20.0%)
Complications	17 (20.0%)
Complete recovery	16 (18.8%)
Deceased	13 (15.3%)
Complications	
None	54 (63.5%)
Amputation	10 (11.8%)
Sepsis	8 (9.4%)
Wound infection	7 (8.2%)
Organ failure	6 (7.1%)
POCUS assessment at three weeks	
Persistent changes	31 (36.5%)
Resolved	28 (32.9%)
Partially resolved	26 (30.6%)
Patient-reported outcomes	
Symptom relief score of 6-10	45 (52.9%)
Wound healing score of 6-10	49 (57.6%)

**Figure 2:**
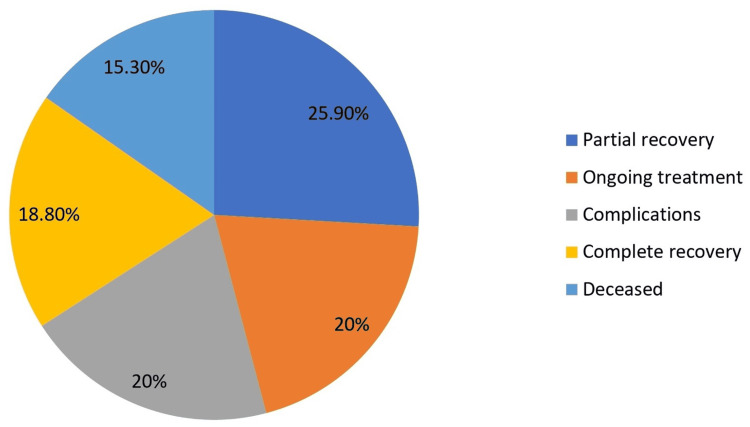
Pie chart showing clinical outcomes at three weeks

The clinical outcomes at three weeks post-intervention are illustrated in Figure 2, showing the distribution of recovery status and mortality.

**Figure 3:**
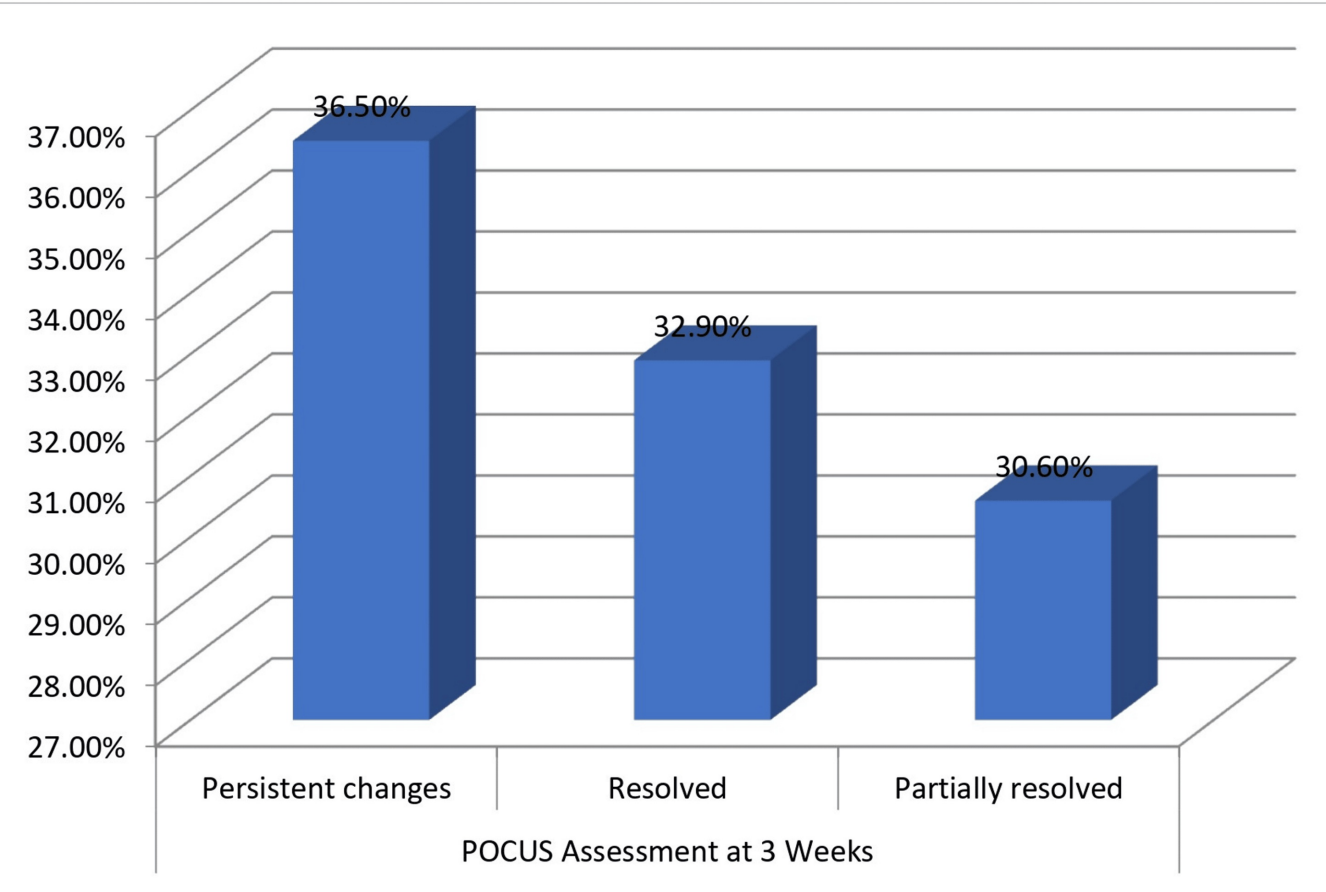
Bar graph showing POCUS assessment at three weeks POCUS: point-of-care ultrasound

Figure 3 demonstrates the POCUS assessment findings at three weeks, indicating the proportion of patients with persistent changes, partial resolution, and complete resolution.

## Discussion

Our study demonstrated that point-of-care ultrasound (POCUS) is a highly sensitive diagnostic tool for necrotizing fasciitis (NF), with 97.6% positivity and specific sonographic markers, including fluid collection, 77.6%; the loss of vascularity, 65.9%; and fascial thickening, 52.9%. The high sensitivity of POCUS for fascial thickening (97.1%) and fluid collection (92.5%) aligns with the findings of Yen et al. [6] and Castleberg et al. [7], while the lower sensitivity for subcutaneous gas (66.2%) mirrors observations by Kim et al. [10]. The demographic profile in our study (majority aged 41-60 years, 62.4% men, and predominantly lower limb involvement) corresponds with findings by Goh et al. [2], Hsiao et al. [11], and Wong et al. [12], while our laboratory findings (elevated WBC count, CRP, and creatinine) and LRINEC scores (50.6% with scores of >8) align with observations by Tan et al. [13] and Wong and Wang [14].

The clinical trajectory in our cohort showed varied outcomes at one week (36.5%, critical; 30.6%, stable; and 29.4%, improved) and three weeks (25.9%, partial recovery; 18.8%, complete recovery, with a 15.3% mortality rate). This mortality aligns with Bocking et al. [15] but is lower than reported by Arif et al. [16], potentially reflecting advancements in diagnostic approaches. Our findings regarding multiple debridements (43.5% requiring three procedures) and our complication rates, including amputation (11.8%), are comparable to Chen et al. [17]. The usefulness of POCUS for monitoring disease progression, as evidenced by our three-week assessment (36.5%, persistent changes; 32.9%, resolution), mirrors findings by Lin et al. [18], while the approach of serial examinations to detect subclinical progression aligns with Khan et al. [19]. The trend toward poorer outcomes with higher LRINEC scores (18.6% mortality with scores of >8 versus 8.6% with scores of <5), though not statistically significant, corresponds with Al-Qurayshi et al., who demonstrated that LRINEC scores of >6 were associated with increased mortality risk [20]. Our study highlighted special considerations for specific populations, including diabetics, the elderly, and immunocompromised patients, as discussed by Cheng et al. [21], Hua et al. [22], and Martinschek et al. [23], respectively, while the comparison of POCUS with alternative imaging modalities (CT and MRI) revealed comparable or superior diagnostic performance, consistent with meta-analyses by Megas et al. [24].

The limitations of our study include its single-center design, relatively small sample size, the operator-dependent nature of ultrasound interpretation, and inter-rater variability. Future directions should include multicenter validation studies and the integration of artificial intelligence algorithms for automated interpretation.

Based on our findings and integration with existing literature, we recommend incorporating POCUS into initial assessment protocols for suspected NF, focusing on key sonographic markers, utilizing serial examinations for monitoring disease progression, combining POCUS findings with LRINEC scores and clinical assessment, implementing standardized training programs, and developing institutional protocols integrating POCUS into diagnostic algorithms. POCUS’s immediate availability, non-invasive nature, and repeatability are valuable in assessing and monitoring this life-threatening condition, potentially expediting diagnosis, guiding surgical interventions, and improving clinical outcomes.

## Conclusions

This study highlights the high sensitivity of point-of-care ultrasound (POCUS) for detecting fascial thickening and fluid collection in patients with necrotizing fasciitis, demonstrating its potential as a valuable diagnostic tool. With sensitivities of 97.1% and 92.5%, respectively, these findings suggest that POCUS can be a reliable and rapid modality for the early identification of key signs of necrotizing fasciitis, enabling quicker decision-making and intervention. The high specificity across all parameters further supports POCUS as an effective screening tool, particularly in resource-limited settings where access to advanced imaging may be restricted. By integrating POCUS into early evaluation protocols, clinicians may improve diagnostic efficiency and patient outcomes, particularly in critically ill patients who cannot be safely transported for more invasive procedures. Larger multicenter studies are needed to validate standardized POCUS protocols and establish definitive criteria for the early, accurate diagnosis of this life-threatening condition.
